# Measuring calcium content in plants using NEXAFS spectroscopy

**DOI:** 10.3389/fpls.2023.1212126

**Published:** 2023-08-16

**Authors:** Sintu Rongpipi, William J. Barnes, Oskar Siemianowski, Joshua T. Del Mundo, Cheng Wang, Guillaume Freychet, Mikhail Zhernenkov, Charles T. Anderson, Esther W. Gomez, Enrique D. Gomez

**Affiliations:** ^1^ Department of Chemical Engineering, The Pennsylvania State University, University Park, PA, United States; ^2^ Department of Biology, The Pennsylvania State University, University Park, PA, United States; ^3^ Advanced Light Source, Lawrence Berkeley National Laboratory, Berkeley, CA, United States; ^4^ National Synchrotron Light Source II, Brookhaven National Laboratory, Upton, NY, United States; ^5^ Department of Biomedical Engineering, The Pennsylvania State University, University Park, PA, United States; ^6^ Department of Materials Science and Engineering and Materials Research Institute, The Pennsylvania State University, University Park, PA, United States

**Keywords:** primary cell wall, inductively coupled plasma mass spectrometry, transmission NEXAFS, fluorescence yield NEXAFS, onion epidermis, Arabidopsis hypocotyl

## Abstract

Calcium is important for the growth and development of plants. It serves crucial functions in cell wall and cell membrane structure and serves as a secondary messenger in signaling pathways relevant to nutrient and immunity responses. Thus, measuring calcium levels in plants is important for studies of plant biology and for technology development in food, agriculture, energy, and forest industries. Often, calcium in plants has been measured through techniques such as atomic absorption spectrophotometry (AAS), inductively coupled plasma–mass spectrometry (ICP-MS), and electrophysiology. These techniques, however, require large sample sizes, chemical extraction of samples or have limited spatial resolution. Here, we used near-edge X-ray absorption fine structure (NEXAFS) spectroscopy at the calcium L- and K-edges to measure the calcium to carbon mass ratio with spatial resolution in plant samples without requiring chemical extraction or large sample sizes. We demonstrate that the integrated absorbance at the calcium L-edge and the edge jump in the fluorescence yield at the calcium K-edge can be used to quantify the calcium content as the calcium mass fraction, and validate this approach with onion epidermal peels and ICP-MS. We also used NEXAFS to estimate the calcium mass ratio in hypocotyls of a model plant, *Arabidopsis thaliana*, which has a cell wall composition that is similar to that of onion epidermal peels. These results show that NEXAFS spectroscopy performed at the calcium edge provides an approach to quantify calcium levels within plants, which is crucial for understanding plant physiology and advancing plant-based materials.

## Introduction

Calcium is an essential element for the normal growth and development of plants. It performs multiple functions, including acting as a structural component of cell walls and cell membranes, as well as an intracellular secondary messenger (Demarty et al., 1984; Hocking et al., 2016; Thor, 2019). Calcium ions increase cell wall cohesion and rigidity by crosslinking the negatively charged carboxyl groups of de-esterified pectin (Demarty et al., 1984; Cleland et al., 1990; Thor, 2019). They also regulate the structure and function of cell membranes by stabilizing lipid bilayers through phospholipid binding and controlling membrane permeability (Demarty et al., 1984; Hepler, 2005; Thor, 2019). Furthermore, calcium serves as a secondary messenger in signaling events relevant to physiological, developmental, and environmental cues related to nutrition and immunity (Thor, 2019).

Calcium content in plants ranges between 0.005 to 5% of dry weight, depending on the plant species, tissue type, and environmental conditions (Jones and Lunt, 1967; Demarty et al., 1984; Thor, 2019); 60-75% of calcium is localized in cell walls (Rossignol et al., 1977; Demarty et al., 1984; Pegg et al., 2019). Calcium deficiency is associated with a number of defects in plants, such as blackening and curling of leaves leading to necrosis and cessation of growth, poor root development, blossom end rot, bitter pit, fruit cracking, poor fruit storage, and water soaking (Jones and Lunt, 1967; White and Broadley, 2003; Hepler, 2005). Given the essential structural and functional roles of calcium in plants, measuring calcium levels in plants is useful not only for studies of plant structure and function, but also for advances in plant-based industries.

Calcium content in plant samples can be determined either through techniques that directly measure calcium or through binding of compounds to calcium that can then be measured. Detection of calcium can be achieved through atomic absorption spectrophotometry (AAS) (Virk and Cleland, 1988; Virk and Cleland, 1990), approaches based on inductively coupled plasma–mass spectrometry (ICP-MS) (Masson et al., 2010; Minocha et al., 2015; Bertin et al., 2016), and electrophysiology (microelectrode) (Miller and Sanders, 1987; Felle, 1988) methods. Binding synthetic chemicals or protein-based dyes to calcium can also facilitate detection, often by fluorescence imaging or colorimetric probes (Williamson and Ashley, 1982; Tsien et al., 1984; Grynkiewicz et al., 1985; Keith et al., 1985; Gilroy et al., 1986; Mcainsh et al., 1990). In addition, nanoparticles and magnetic resonance imaging (MRI) have been proposed for calcium detection in plants (Van Dusschoten et al., 2016; Dixit et al., 2021). These techniques, however, present various challenges, including the large sample size required when relying on direct detection, requirements for chemical extraction, reliance on the diffusion of probe compounds, and limited spatial resolution in many cases (beyond manual sectioning). Additionally, microelectrode techniques are technically difficult to apply, and have slow response times and low sensitivity (Felle, 1989).

A few optical spectroscopic techniques, such as Visible - near IR spectroscopy (Petisco et al., 2005; Li et al., 2019) and laser induced breakdown spectroscopy (Pouzar et al., 2009; Nunes et al., 2010), have demonstrated direct measurement of calcium content in plant samples without requiring chemical extraction. These methods, however, required grinding plant tissues into powder form and later packed into a pellet form or in a closed sample cup for analysis. Often, a dried plant sample size of ~500 mg is required for forming a pellet (Nunes et al., 2010), which can be tedious to obtain from certain types of plant tissue.

X-ray spectroscopy, such as Near-edge X-ray absorption fine structure (NEXAFS) or X-ray absorption near edge structure (XANES) can also, in principle, quantitatively detect calcium in intact plant tissues. NEXAFS probes the X-ray absorption or electron or photon emission associated with electronic transitions, from core-level to unoccupied energy levels, that occur when the energy of incident X-rays is near the absorption edge of an element present in the sample (Leri et al., 2006; Watts et al., 2006; Stöhr, 2013). The chemical specificity of core-level electronic transitions makes the technique sensitive only to the element of interest, and this approach negates interference from other components in the sample. NEXAFS does not require large sample size, requires little sample preparation, and can spatially resolve calcium composition when a focused probe is used (X-ray probes are approaching about 20 nm in diameter (Jefimovs et al., 2007; Watts et al., 2012; Collins and Gann, 2022)). This makes NEXAFS advantageous for the analysis of chemically heterogeneous samples, such as biological assemblies. The magnitude of jump in absorption at the edge in a NEXAFS spectrum, called an edge jump, is representative of the content of the absorbing element. This technique has been used to quantify chlorine and bromine in soft organic samples (Leri et al., 2006; Leri and Ravel, 2014), but its ability to probe calcium content in biological samples has not been fully explored.

In this work, we use NEXAFS spectroscopy obtained at the calcium L-edge through measurements of the X-ray absorption to quantify the calcium to carbon mass ratio in onion epidermal peels and dark grown (etiolated) hypocotyls of *Arabidopsis thaliana*. We also demonstrate that collecting NEXAFS spectra using the fluorescence yield can reveal calcium content, with the caveat that in this mode NEXAFS is surface-sensitive (Watts et al., 2006). We normalize NEXAFS spectra at the calcium L-edge by the edge jump at the carbon K-edge to account for the effects of sample thickness and obtain a measure of calcium content. Absorbance values from transmission NEXAFS integrated over energies 345.0 eV to 355.0 eV at the calcium L-edge and measurements of calcium mass fraction in onion epidermis made using ICP-MS are compared to obtain a calibration curve for determining the calcium mass fraction in plant samples from NEXAFS. This calibration curve was then used to estimate the calcium mass fraction in the top, middle and bottom of Arabidopsis hypocotyls solely from NEXAFS data, demonstrating the utility of this approach.

## Materials and methods

### Sample preparation

#### Onion epidermal cell wall

The periclinal wall from the abaxial surface of scales of white onion (*Allium cepa* L. cometa) bulbs, obtained from local grocery stores, was prepared as previously described (Zhang and Cosgrove, 2017). All peels were rinsed with deionized (DI) water. Water-rinsed onion peels were used as unextracted samples. For calcium-treated samples, onion peels were incubated in 2 mM CaCl_2_, 20 mM Tris buffer (pH 9.5) for 16 h at 37°C with gentle shaking at 50 rpm. After incubation, samples were rinsed with DI water.

For transmission NEXAFS measurements, hydrated onion peels were mounted on 50 nm thick Si_3_N_4_ windows with a 5 mm × 5 mm silicon frame (Norcada NX5100A) and then air dried. For fluorescence NEXAFS measurements, hydrated onion peels were mounted as free-standing films on a sample bar and then air dried.

#### Hypocotyls of *Arabidopsis thaliana*

Seedlings of *Arabidopsis thaliana* Columbia (Col-0) ecotype were sterilized in 30% bleach solution containing 0.1% (w/v) sodium dodecyl sulfate (SDS) for 20 minutes with occasional mixing, washed in sterile water four times, resuspended in 0.15% agar (Sigma), and stored at 4°C for 2-7 days for vernalization. Seeds were sown on ½ Murashige and Skoog (MS) plates and wrapped in two layers of aluminum foil to induce etiolation and grown in a 22°C chamber for 6 days before harvesting, flash-freezing, and storage at -80°C until sample preparation. MS plates contained 2.2 g/L MS salts (Caisson Laboratories), 0.6 g/L 2-N-morpholino-ethanesulfonic acid (MES; Research Organics), and 0.8% (w/v) agar-agar (Research Organics) at pH 5.6.

Hypocotyls of 6-day-old dark grown *Arabidopsis thaliana* seedlings were rinsed with DI water. For transmission NEXAFS measurements, 20-30 hydrated, non-overlapping hypocotyls were mounted flat on 50 nm thick Si_3_N_4_ windows with a 5 mm × 5 mm silicon frame (Norcada NX5100A) and then air dried. For fluorescence NEXAFS measurements, 20-30 hydrated hypocotyls were mounted as free-standing bundles on a sample bar and then air dried.

### Acid digestion of cell walls for ICP-MS measurements

#### Acid digestion of onion cell walls

Onion peels rinsed with DI water were air dried for a minimum of 3 days and then digested with acid using the following protocol. Fifty milligrams of dried onion cell wall were incubated with 25% (v/v) HNO_3_ in a polytetrafluoroethylene (PTFE) vial at room temperature for 1 h. The sample was then incubated for 1 h on a hotplate at 60°C, after which the temperature was increased to 100-115°C and the sample was allowed to flux overnight. The following morning, 4-5 drops of ultra-pure H_2_O_2_ were added, and the solution was allowed to react at 60°C for 1-2 h. The temperature was then increased to 100-115°C and the solution was evaporated. Once dried, 1.5 mL water and 0.5 mL of 25% (v/v) HNO_3_ were added, and the sample was again allowed to flux overnight on a hotplate. Then the vial was cooled and weighed to determine the mass of the final solution. 0.8 mL of the solution was then diluted to 10 mL with 2% (v/v) HNO_3_ prior to analysis.

#### Acid digestion of hypocotyls of *Arabidopsis thaliana*

6-day-old dark grown Col-0 hypocotyls were used for the analysis. Roots and cotyledons were cut off and hypocotyls were washed in DI water 3 times and then dried at 60°C for 48 h. Following drying, the dry weight was measured. Samples were then put into an acid-cleaned PTFE vial, and 2 mL of 4 N double distilled HNO_3_ was added, and the sample was heated overnight at 115°C on a hotplate. Next, 4 drops of ultra-pure H_2_O_2_ were added, and the solution was evaporated on a hotplate at 90°C. Then, 5 mL of 4 N double distilled HNO_3_ was added, and the sample was again heated overnight at 115°C. Similar to above, these samples were also then diluted with 2% (v/v) HNO_3_ prior to analysis.

### Inductively coupled plasma mass spectrometry (ICP-MS) measurements

Calcium mass fraction in the cell wall samples were determined by Inductively Coupled Plasma Mass Spectrometry (ICP-MS) with Collision Cell Technology (CCT) using the Laboratory for Isotopes and Metals in the Environment (LIME) facilities at The Pennsylvania State University. Acid digested cell wall samples were run on a Thermo Fisher Scientific Icap RQ (ICP-MS), which has an instrumental detection limit of 51.4 μg/mL for calcium, using external standard NIST SRM 1640a (Trace Elements in Water).

### Transmission NEXAFS

Transmission NEXAFS spectra were collected at beamline 11.0.1.2 at the Advanced Light Source (ALS), Lawrence Berkeley National Laboratory. Details regarding the beamline optics have been reported previously (Gann et al., 2012). The angle between the sample to incident X-ray beam was maintained at 90°. Spectra from 270 eV to 375 eV (energy resolution ~ 100 meV) covering the carbon K-edge and calcium L-edge were collected within a single scan. Transmitted X-ray intensity was recorded using a photodiode detector. NEXAFS spectra were normalized with respect to direct beam flux and blank substrate absorption. The direct beam flux includes response from a photodiode inside the scattering chamber and the drain currents from a gold mesh upstream from the sample (Gann et al., 2012; Ferron et al., 2020). This double normalization approach mitigates the effect of the carbon dip from carbon contamination in upstream optics on the collected carbon NEXAFS data.

For background correction, a line was fitted to the pre-edge region (energies of 340.0 to 342.0 eV) of NEXAFS absorbance spectra, and this line was then subtracted from the rest of the data. This choice of background subtraction is based on the resulting NEXAFS spectra after subtraction with several baseline fits (**Figures S1A–D**) from pre-edge and post-edge regions as discussed in **Supplemental Section 2**.

### Fluorescence yield (FY) NEXAFS

Fluorescence NEXAFS spectra were collected at the Soft Matter Interfaces (SMI) beamline of the National Synchrotron Light Source II (NSLS-II) at Brookhaven National Laboratory (BNL) (Zhernenkov et al., 2014). Samples were measured in transmission mode, mounted perpendicular to the beam. Spectra from 4020 eV to 4150 eV (energy resolution ~ 0.4 eV) covering the calcium K-edge were collected within a single scan. The X-ray fluorescence intensity was recorded using a Pilatus 300K-W detector, consisting of 0.172 mm square pixels in a 1475 × 195 array, mounted at a fixed distance of 0.275 m from the sample position. To limit contamination of the fluorescence yield intensity by the scattering signal, the Pilatus detector was moved horizontally on a fixed arc to a high angle of 52 degrees. Moreover, the beam is fully polarized horizontally at the SMI beamline and because the scattering decreases as cos^2^θ (θ being the angle between the polarization plane and detector plane), the contributions from scattering to the measured intensity are ensured to be low. Measurements were performed in a vacuum chamber and NEXAFS spectra were normalized with respect to direct beam flux, measured on diamond beam position monitors (Pandolfi et al., 2018).

### 2F4 Immunolabeling

Immunolabeling with the 2F4 primary antibody was performed on 6-d-old etiolated seedlings according to a previously reported protocol (Rui et al., 2017) with minor modifications. A solution of 0.5% (w/v) Fast Green FCF (w/v; Electron Microscopy Sciences, Hatfield, PA) in ethanol was included in the final ethanol dehydration step (100% ethanol) to help locate hypocotyl samples embedded in LR White resin. After immunolabeling, 0.01% (w/v) Fluorescent Brightener 28 (FB28, the fluorophore in Calcofluor White M2R) was applied for 10 min to completely label cell walls. Immunolabeled and FB28-stained sections were imaged as Z-stacks on a Zeiss Axio Observer microscope with a Yokogawa CSU-X1 spinning disk head and a 20× 0.5 NA air objective using a 405 nm excitation laser and 450/50 nm emission filter for FB28, and a 488-nm excitation laser and a 525/50-nm emission filter for detection of the Alexa Fluor 488-labeled secondary antibody bound to the 2F4 primary antibody.

### Statistical analysis

All experimental results are from at least three biological replicates. The data are presented as a mean ± standard error of the mean. Statistical analyses were performed using student’s t-test and samples were also compared by one-way ANOVA, followed by the Tukey–Kramer *post hoc* test (p< 0.05) for multiple comparisons. As an additional method to establish whether calcium composition differed between samples, the 95% confidence interval of the differences of the mean of the calcium mass fraction in Arabidopsis hypocotyls obtained from a calibration curve and from ICP-MS was calculated. Confidence intervals of the differences in means that exclude zero support the hypothesis that calcium mass fraction varies between samples being considered (Altman and Krzywinski, 2017).

## Results

**Figure 1:**
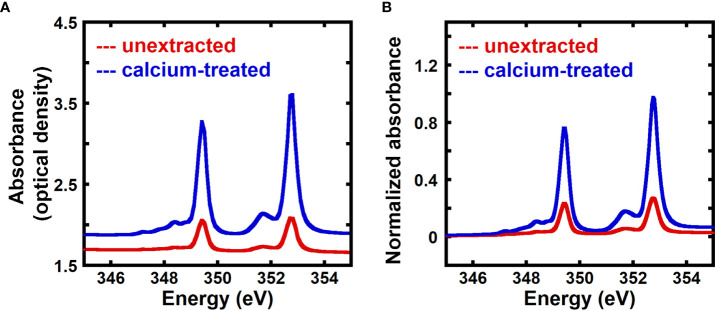
Calcium-treated onion epidermal cell walls show enhanced NEXAFS signal at the calcium edge. **(A)** Absorbance in optical density obtained from transmission near edge X-ray absorption fine structure (NEXAFS) spectroscopy near the calcium L_3,2_ edge of unextracted and calcium-treated onion 5^th^ scale epidermal cell wall. **(B)** Absorbance at the calcium L_3,2_ edge of unextracted and calcium-treated onion 5^th^ scale epidermal cell wall background corrected using the pre-edge region (340.0 eV to 342.0 eV) as described in the Methods Section and normalized by the carbon edge step jump (Absorbance_325.0 eV_ – Absorbance_270.0 eV_).

Previous work has shown that transmission NEXAFS near the calcium L_3,2_ edge can detect the presence of calcium in onion cell walls using native (unextracted) samples or samples treated with 2mM CaCl_2_, although no quantification was performed (Ye et al., 2018). Here, we use transmission NEXAFS measurements of the onion 5^th^ scale to quantitatively measure the calcium to carbon mass ratio in the cell wall, and assume that this ratio equals the mass fraction of calcium given the small calcium content by mass. **Figure 1A** shows the X-ray absorbance as optical density of unextracted and calcium-treated onion cell wall. The energy range for the calcium L_3,2_ edge is 330.0 to 360.0 eV (Chen et al., 2015; Ye et al., 2018), and the peaks in the NEXAFS spectra at 349.3 eV and 352.6 eV are due to 2p → 3d Ca L_3_ and L_2_ transitions, respectively (Naftel et al., 2001; Ingham et al., 2015; Ye et al., 2018). The NEXAFS absorbance at the Ca edges is higher for calcium-treated cell wall when compared to unextracted samples.

Because the NEXAFS intensity is proportional to the total amount of absorbers, quantitative comparisons require an accurate estimate of sample thickness (Buckley et al., 1995; Buckley and Zhang, 1998; Leri and Ravel, 2014). We can account for the effects of sample thickness by normalizing NEXAFS intensities by the mass thickness calculated at the carbon edge. Transmission of X-rays through a material of density *p* and thickness *t* can be described by (Buckley and Zhang, 1998):


(1) $$I=\;I_{0\;}\exp\left(-\mu \rho t\right)$$

where *I* is the transmitted X-ray intensity, *I_0_* is the incident X-ray intensity, and *μ* is the mass absorption coefficient at the particular X-ray energy. The product ρ*t* is often called the mass thickness. Equation (1) can be expressed in terms of absorbance, $Abs=\;-\text{ln}(\frac{I}{I_{0}})$ as


(2) Abs= μρt

If a sample contains an element *X* in a matrix composed of n other elements *Y_l_, Y_2_,…, Y_n_*, with densities and thicknesses of *ρ_X_*, *ρ_Y1_, ρ_Y2_, ···, ρ_Yn_* and *t_X_*, *t_Y1_, t_Y2_, …, t_Yn_* respectively, we can write (Buckley, 1995):


(3) $$Abs=\;\mu_{X}\rho_{X}t_{X}+\;\sum_{i=1}^{n}\mu_{Yi}\rho_{Yi}t_{Yi}$$

The difference in absorbance for two energies 1 and 2 can be written as:


(4) $$\Delta Abs=Abs_{2}-\;Abs_{1}=\left(\mu_{X2}-\;\mu_{X1}\right)\rho_{X}t_{X}+\;\sum_{i=1}^{n}(\mu_{Yi2}-\mu_{Yi1})\rho_{Yi}t_{Yi}$$

such that the mass thickness of element *X* can be determined from:


(5) $$\rho_{X}t_{X}=\;\frac{\Delta Abs\;–\;\sum_{i=1}^{n}(\mu_{Yi2}-\mu_{Yi1})\rho_{Yi}t_{Yi}\;}{\left(\mu_{X2}-\;\mu_{X1}\right)}$$

Near an elemental edge, where energy 2 is post-edge and energy 1 is pre-edge, we can assume the difference in absorption of the element of interest is much larger than the difference in mass absorption coefficient of all other elements, such that $\Delta Abs\gg \;\sum_{i=1}^{n}(\mu_{Yi2}-\mu_{Yi1})\rho_{Yi}t_{Yi}$ . This inequality is satisfied when the composition of the element of interest is high, *e.g.*, at the carbon edge for plant cell walls. We thus determine the mass thickness of carbon *ρ_c_t_c_* as:


(6) $$\rho_{C}t_{C}\approx \;\frac{\left(Abs_{2}-\;Abs_{1}\right)\;}{\left(\mu_{C2}-\;\mu_{C1}\right)}=\;\frac{\Delta Abs_{C}\;}{\Delta \mu_{C}}$$

Given the large difference in absorption before and after the carbon edge, we use the absorbance at a pre-edge energy of 270.0 eV and post-edge energy of 325.0 eV to calculate the carbon edge jump $\left(\Delta Abs_{C}=\;Abs_{325.0eV}-Abs_{270.0eV}\right)$ . This avoids distortions of NEXAFS spectra from the monochromator bandpass that can occur near sharp peaks (Buckley, 1995).

As seen in **Figure 1B**, the pre-edge to post-edge jump is small at the calcium L-edge, especially when compared to the absorption at energies on the Ca edge (e.g., L_3_: 349.3 eV and L_2_: 352.6 eV). We therefore integrate the absorbance between *E_1_ =* 345.0 eV and *E_2_ =*355.0 eV to increase the accuracy of our measurements of calcium content, as similarly done for concentration maps from scanning X-ray microscopy data (Buckley, 1995). Integrating the absorbance over multiple energies can be described by modifying equation 6 to yield the mass thickness of calcium:


(7) $$\rho_{Ca}t_{Ca}=\;\frac{\int_{E_{1}}^{E_{2}}Abs_{Ca}dE}{\int_{E_{1}}^{E_{2}}\mu_{Ca}dE}$$

The monochromator bandwidth at the ALS beamline 11.0.1.2 is 0.1 eV and the widths of calcium resonance peaks are greater than 0.3 eV. Because of this, we assume that the distortion to NEXAFS spectra due to the monochromator band pass is minimal at the Ca edge (Buckley and Zhang, 1998). In addition, such effects should have minimal consequences on our measurements because we integrate over a broad energy range (10 eV).

We can account for differences in sample thickness by normalizing by the carbon mass thickness (obtained from equation 6), which results in the calcium to carbon mass ratio (*ζ_Ca_*) of the sample, as given below:


(8) $$\zeta_{Ca}=\;\frac{Mass\;of\;calcium}{Mass\;of\;carbon}=\frac{\rho_{Ca}t_{Ca}}{\rho_{C}t_{C}}=\;\frac{\int_{E_{1}}^{E_{2}}Abs_{Ca}dE}{\Delta Abs_{C}}\;\times \;\frac{\Delta \mu_{C}}{\int_{E_{1}}^{E_{2}}\mu_{Ca}dE}$$

Equation (8) shows that *ζ_Ca_* can be obtained from $\frac{\Delta \mu_{C}}{\int_{E_{1}}^{E_{2}}\mu_{Ca}dE}$ and the integrated calcium edge absorbance normalized by the carbon edge jump $\left(\frac{\int_{E_{1}}^{E_{2}}Abs_{Ca}dE}{\Delta Abs_{C}}\right)$ . Thus, to obtain an absolute value of *ζ_Ca_*, the mass absorption coefficients at the calcium and carbon edges are needed (Buckley, 1995). Alternatively, we can obtain the relative calcium to carbon mass ratio in two plant samples of similar chemical composition by comparing the integrated absorbance spectra at the calcium L-edge normalized by the edge jump at the carbon K-edge while assuming that $\frac{\Delta \mu_{C}}{\int_{E_{1}}^{E_{2}}\mu_{Ca}dE}$ is invariant between samples. Given that plant cell walls are mostly carbon, the calcium to carbon mass ratio is approximately the same as the calcium mass fraction (*w_Ca_* = calcium mass/total mass), and the calcium concentration is given by the product of *w_Ca_* and mass density of the sample.

**Figure 1B** shows absorbance spectra of unextracted and calcium-treated cell walls, background corrected using the pre-edge region (340.0 eV to 342.0 eV), as described in the Methods, and normalized by the carbon edge jump. Normalized absorbance at the calcium L-edge is enhanced for calcium-treated cell wall in comparison to unextracted cell wall, suggesting that transmission NEXAFS spectra are sensitive to the concentration of calcium in the cell wall and can be used for relative quantification of calcium.

**Figure 2:**
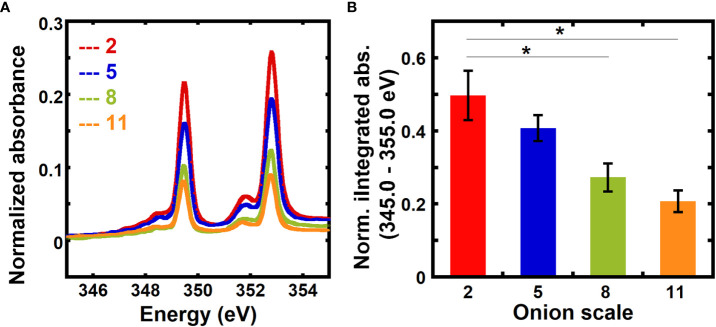
Absorbance at the calcium edge varies with the age of onion scales. **(A)** Absorbance near the calcium L_3,2_ edge of unextracted onion 2^nd^, 5^th^, 8^th^, and 11^th^ scale epidermal cell walls. Spectra are corrected by a linear background obtained from the pre-edge (340.0 to 342.0 eV) as described in the Methods and are normalized by the carbon edge step jump (Absorbance_325.0 eV_ – Absorbance_270.0 eV_). **(B)** Absorbance integrated from 345 eV to 355 eV and normalized by the carbon edge jump for unextracted onion 2^nd^, 5^th^, 8^th^, and 11^th^ scale epidermal cell walls. Error bars represent standard error of the mean and asterisks indicate significant differences (n ≥ 3, * p < 0.05).

The relative calcium mass fractions in 2^nd^, 5^th^, 8^th^, and 11^th^ onion scales, which represent different developmental ages, were obtained using transmission NEXAFS measurements. The numbering of the onion scales reflects the order of their formation during bulb development, where the 2^nd^ scale is the oldest and the 11^th^ scale is the youngest among the scales examined (Suslov et al., 2009; Kafle et al., 2014; Zhang et al., 2014). **Figure 2A** shows normalized absorbance spectra for different onion scales in the energy range 345.0 eV to 355.0 eV. The normalized absorbance spectra for different onion scales for the entire calcium L-edge (330.0 eV to 360.0 eV) are shown in **Figure S2A**. Normalized absorbances at on-edge energies (L_3_: 349.3 eV and L_2_: 352.6 eV) are higher for older 2^nd^ and 5^th^ scales than for younger 8^th^ and 11^th^ scales.

**Figure 2B** shows a comparison of background-corrected absorbance normalized by the carbon edge jump and integrated from 345 eV to 355 eV for onion scales. Older 2^nd^ and 5^th^ scales show higher normalized integrated absorbance than younger 8^th^ and 11^th^ scales. No significant difference in the normalized integrated absorbance is found between 2^nd^ and 5^th^ scales. Significantly higher normalized integrated absorbance seen in older onion scales as compared to younger scales suggests that the calcium mass fraction in primary cell walls increases as a function of developmental age of the tissue.

**Figure 3:**
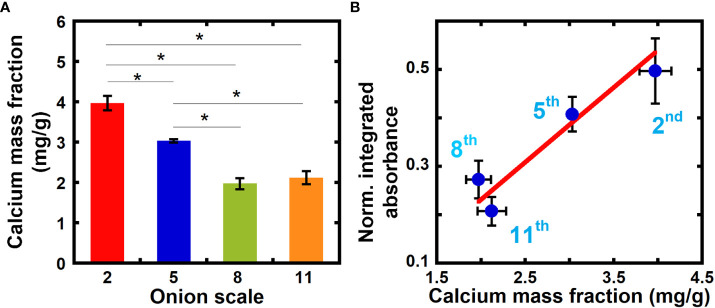
Inductively Coupled Plasma Mass Spectrometry (ICP-MS) reveals higher calcium mass fraction in older onion scales when compared to younger scales. **(A)** Calcium mass fraction in unextracted onion 2^nd^, 5^th^, 8^th^, and 11^th^ scale epidermal cell walls obtained from ICP-MS. Error bars represent standard error of the mean and asterisks indicate significant differences (n ≥ 3, * p < 0.05). **(B)** Correlation between normalized integrated absorbance (345.0 to 355.0 eV) and calcium mass fraction in onion epidermal cell wall obtained from ICP-MS from unextracted onion epidermal cell walls. Line denotes a weighted linear fit between the NEXAFS absorbance ratio (Y) and ICP calcium mass fraction in mg/g (X). Y = (-0.08 ± 0.11) + (0.15 ± 0.04) X, R^2 =^ 0.91.

We correlate our measurements of relative calcium composition from NEXAFS spectra to values of the calcium mass fraction from ICP-MS, and in this way obtain a quantitative measure of $\frac{\Delta \mu_{C}}{\int_{E_{1}}^{E_{2}}\mu_{Ca}dE}$ in equation 8. ICP-MS is a highly sensitive elemental analysis technique that uses mass spectrometry to measure samples ionized with high-temperature plasma (Houk, 1986; Pröfrock and Prange, 2012). **Figure 3A** shows the calcium mass fraction in 2^nd^, 5^th^, 8^th^, and 11^th^ scale onion peels obtained from ICP-MS. The calcium mass fraction in older 2^nd^ and 5^th^ scales is significantly higher as compared to younger 8^th^ and 11^th^ scales. No significant difference in calcium mass fraction is seen between the two younger scales (8^th^ and 11^th^).

**Figure 3B** plots the normalized integrated absorbance at the Ca L-edge energies 345.0 eV to 355.0 eV ( $\frac{\int_{E_{1}}^{E_{2}}Abs_{Ca}dE}{\Delta Abs_{C}}\;$ in equation 8) against the calcium mass fraction obtained from ICP-MS for each measured onion scale. A linear relationship exists between the normalized integrated absorbance and calcium mass fraction from ICP-MS with an R-squared value of 0.91 (Pearson correlation coefficient between the two quantities is 0.95). We can use this linear fit to estimate the calcium mass fraction in primary cell walls directly from NEXAFS data, by assuming that the carbon density is similar across cell walls from different sources. For example, our linear fit yields a calcium mass fraction of 9.85 mg/g for 5^th^ scale onion peels treated with calcium (NEXAFS data shown in **Figure 1**), which is higher than unextracted samples, as expected.

Given that onion and Arabidopsis have similar compositions of primary cell walls (Redgwell and Selvendran, 1986; Ryden et al., 2003), we also estimated calcium mass fraction in Arabidopsis samples from NEXAFS data using the calibration curve from onion. We collected calcium L-edge transmission NEXAFS data of hypocotyls of 6-day-old dark grown *Arabidopsis thaliana* and used our calibration curve obtained from onion (**Figure 3B**) to estimate the calcium mass fraction in different regions of hypocotyls.

A growth gradient exists along the length of dark grown hypocotyls (Refrégier et al., 2004). Each hypocotyl was ~15 mm long and we denote the region 0 to 5 mm from the cotyledons as the “top”, the region 5 to 10 mm from the cotyledons as the “middle”, and the region 10 to 15 mm from the cotyledons (near the root) as the “bottom”. Tissue age as a function of growth increases when moving from the top to the bottom along the hypocotyl. NEXAFS spectra of the three regions along the hypocotyls were measured separately and calcium mass fractions were estimated in each region using the linear relationship developed in **Figure 3B**.

**Figure 4:**
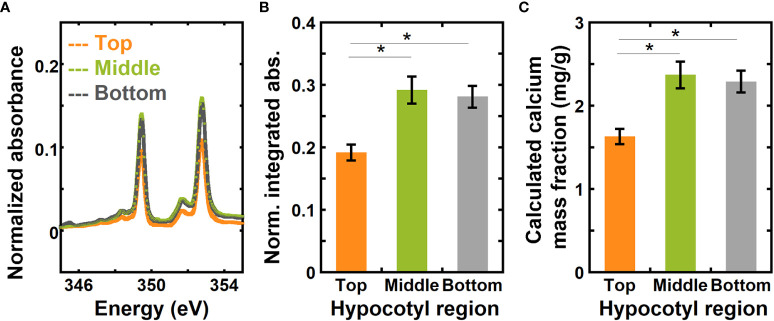
NEXAFS absorbance at the calcium L_3,2_ edge shows higher calcium mass fraction in the middle and bottom regions than in the top region of 6-day-old hypocotyls of *Arabidopsis thaliana*. **(A)** Absorbance at the calcium L_3,2_ edge of top, middle, and bottom regions of 6-day-old hypocotyls of *Arabidopsis thaliana*. Spectra are corrected by a linear background obtained from the pre-edge (340.0 to 342.0 eV) as described in the Methods and normalized by the carbon edge step jump (Absorbance_325.0 eV_ – Absorbance_270.0 eV_). **(B)** Normalized absorbance integrated for energies 345.0 eV to 355.0 eV of top, middle, and bottom regions of hypocotyls. **(C)** Calcium mass fraction in different regions of hypocotyls calculated from the linear relationship between normalized integrated absorbance and calcium mass fraction from ICP-MS of onion scales. Error bars represent standard error of the mean and asterisks indicate significant differences (n = 3, * p < 0.05).

**Figure 4A** shows absorbance spectra of the top, middle, and bottom regions of hypocotyls of 6-day-old dark grown *Arabidopsis thaliana* for the energy range from 345.0 to 355.0 eV that have been background corrected and normalized using a similar approach to the onion samples. Normalized absorbance spectra for the three different regions of hypocotyls for the complete calcium L-edge are shown in **Figure S2B**. **Figure 4B** shows a comparison of normalized integrated absorbance ( $\frac{\int_{E_{1}}^{E_{2}}Abs_{Ca}dE}{\Delta Abs_{C}}\;$ in equation 8) for the top, middle, and bottom regions of Arabidopsis hypocotyls. Using the normalized integrated absorbance and the calibration curve in **Figure 3B**, we estimated calcium mass fraction in the top, middle, and bottom regions of hypocotyls (**Figure 4C**). The normalized integrated absorbance, and thus the calcium mass fraction, is significantly higher in the middle and bottom regions than in the top region.

Obtaining sufficient materials for ICP-MS measurements from each region of 6-day-old Arabidopsis hypocotyls is challenging. Instead, we compared ICP-MS measurements of whole hypocotyls with the average calcium mass fraction estimated from NEXAFS data for the three regions of hypocotyls. The average calcium mass fraction from NEXAFS is 2.09 ± 0.13 mg/g, and from ICP-MS for the whole hypocotyls it is 2.82 ± 0.22 mg/g. The difference between values obtained from NEXAFS and ICP-MS is not statistically significant, as the p-value for a Student’s t-test between the measured and calculated value of calcium concentration is 0.096 (null hypothesis cannot be rejected at the 0.05 significance level). In addition, the 95% confidence interval for the difference of the means is (-0.02, 1.48), which includes zero.

The distribution of calcium along the length of Arabidopsis hypocotyls was also examined through imaging immunolabeled calcium-crosslinked homogalacturonan (HG, the most abundant pectin in primary cell walls). We used 2F4, an antibody that recognizes calcium-crosslinkable HG (Liners et al., 1989), to immunolabel cross sections in the young and rapidly-elongating top region of the hypocotyl as well as in the elongated middle region. We found that there was little 2F4 immunolabeling in the top region (**Figure S3A**), but the 2F4 signal is more widely distributed and intense in the middle region of 6-day-old etiolated Col-0 hypocotyls (**Figure S3B**). These data support our NEXAFS results and suggest that calcium-crosslinkable HG is more prominent in lower regions of etiolated hypocotyls compared to the upper-most region, such that more elongated cell walls of the lower hypocotyl contain more calcium than in the upper region of hypocotyls.

The total thickness of the cell wall sample can be estimated from absorbance at off-edge energies and the mass absorption coefficient of the sample (described in **Supplementary Information Section 1**). We used this to investigate the sensitivity of calcium L-edge NEXAFS to the mass fraction of calcium in the cell wall by normalizing the absorbance spectra by the average absorbance at off-energies 325.0 eV – 330.0 eV, assuming that the mass absorption coefficient and density remain invariant across the different samples tested in this study. Similarly to the NEXAFS spectra normalized by the carbon-edge jump (**Figures 2A, B**), the NEXAFS spectra normalized by absorbance at off-edge energies (**Figures S4A, B**) also show that the calcium mass fraction increases with tissue age in onion peels.

Transmission NEXAFS determines photon absorption by measuring the X-ray beam intensity before and after passing through a sample as the X-ray energy is modulated. This requires some X-ray transmission, placing a limit on sample thickness (a few microns at the carbon edge, depending on the density). Alternatively, we can collect NEXAFS measurements in fluorescence yield (FY) mode, where the total number of photons emitted from the sample per incident photon is measured as a function of incident X-ray energy. FY NEXAFS has been previously used for quantitative analysis of absorber species. For example, it has been used to quantify the concentration of chlorine and bromine in organo-halogens (Leri et al., 2006) and also of bromine in a matrix of poly(acrylic acid, sodium salt) (Leri and Ravel, 2014). NEXAFS in FY and transmission modes are essentially equivalent techniques, because the fluorescence photon yield emitted from the sample is proportional to the absorption coefficient, but with the caveat that emitted photons have a limited escape depth (Kasrai et al., 1996; Stöhr, 2013).

**Figure 5:**
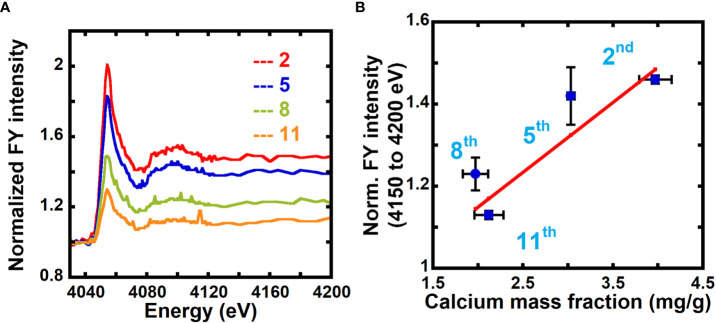
Fluorescence NEXAFS spectroscopy can be used to measure the calcium mass fraction in plant cell walls. **(A)** Fluorescence NEXAFS spectra of unextracted 2^nd^, 5^th^, 8^th^, and 11^th^ onion scales at the calcium K-edge. Spectra are normalized by the average of intensities at energies from 4030 eV to 4040 eV (pre-edge). **(B)** Averaged fluorescence yield (FY) intensity for 4150 eV to 4200 eV (edge jump) normalized by the average intensity for energies 4030 eV to 4040 eV (pre-edge) and calcium mass fraction in onion cell wall obtained from ICP-MS. Line denotes a weighted linear fit between the NEXAFS fluorescence intensity ratio (Y) and the ICP calcium mass fraction (X) in mg/g. Y = (0.17 ± 0.04) + (0.81 ± 0.11) X, R^2^ = 0.81.

In FY NEXAFS, absorption is measured by detecting fluorescent photons on a detector positioned at an angle relative to the surface normal of the sample. While transmission NEXAFS probes the entire thickness of the sample, in FY NEXAFS, the probe depth depends on the mean escape depth of photons from the sample, thereby placing a depth limit of detection. The escape depth for photons for calcium at the Ca L-edge (~350 eV) is ~0.16 to 0.39 μm, and at the Ca K-edge (~4000 eV) it is ~7.3 to 48 μm, depending on whether the energies are at specific resonances or not. Because the thicknesses of our samples are ~1 to 5 μm, FY NEXAFS will probe the entire thickness of the sample at the Ca K-edge, but not at the Ca L-edge. As shown in **Figure 5A** and **Figure S5**, we collected fluorescence yield NEXAFS data of unextracted 2^nd^, 5^th^, and 11^th^ scale and calcium-treated 5^th^ scale at the Ca K-edge to evaluate the feasibility of using FY NEXAFS for quantification of calcium in primary cell walls.

Unlike the soft X-ray regime (X-ray energies less than 1500 eV) where we could access the carbon K-edge, in the tender X-ray regime (X-ray energies between 1500 eV to 8000 eV) we cannot access the carbon absorption edge. Instead of normalizing the FY spectra by the carbon edge jump as done for transmission NEXAFS data, we normalize by the average of FY intensities for off-edge energies from 4030 eV to 4040 eV which, as demonstrated by comparing **Figure S4** and **Figure 2**, yields similar results to normalization by the carbon edge jump. This enables the use of a single tender X-ray instrument instead of requiring an additional experiment at a soft X-ray beamline to acquire carbon edge data.

**Figure S5** shows FY spectra of unextracted and calcium-treated onion 5^th^ scale cell walls normalized by the average of fluorescence intensity over the off-edge energies (4030 eV to 4040 eV). A higher edge jump is seen for the calcium-treated sample as compared to the unextracted sample because of the higher calcium mass fraction in the former. **Figure 5A** shows FY spectra of unextracted and calcium-treated 2^nd^, 5^th^, 8^th^, and 11^th^ onion scales normalized by the average of the fluorescence intensity over the off-edge energies (4030 eV to 4040 eV). The edge jump is higher in older 2^nd^ and 5^th^ scales as compared to younger 8^th^ and 11^th^ scale (**Figure S6**). This indicates higher calcium mass fraction in older tissue as compared to younger tissue, in agreement with results from transmission NEXAFS data (**Figures 2A, B**) and ICP-MS data (**Figure 3A**). We estimate the calcium mass fraction of the sample by taking the edge jump in the FY spectra at the Ca edge, which is determined from the average of fluorescence intensities at post-edge energies (4150 eV to 4200 eV) after normalizing the entire spectra by pre-edge intensities, as shown in equation 9:


(9) $$w_{Ca,\;FY}=\;\frac{Mass\;of\;calcium\;\left(\rho_{Ca}t_{Ca}\right)}{Total\;mass}=\frac{\frac{\Delta Abs_{Ca}}{\Delta \mu_{Ca}}}{\frac{Abs_{off-edge}}{\mu_{sample}}}$$

Because fluorescence yield intensity is proportional to the absorption coefficient, we can write:


(10) $$w_{Ca,FY}\propto \;\frac{FY_{Ca\;edge\;jump}}{FY_{off-edge}}=\frac{FY_{4150-4200\;eV}}{FY_{4030-4040\;eV}}$$

**Figure 5B** plots the normalized FY intensities averaged over post-edge energies (4150 eV to 4200 eV) and the calcium mass fraction from ICP-MS for onion scales. The correlation between the data (Pearson correlation coefficient 0.90) indicates that NEXAFS in fluorescence mode can also be used to quantify the calcium composition in primary cell walls. Calcium-treated samples show a calcium mass fraction of 9.87 mg/g for 5^th^ scale onion peels from FY NEXAFS data (**Figure S5**), which is in good agreement with values obtained from transmission NEXAFS measurements (9.85 mg/g).

## Discussion

NEXAFS measurements in both transmission and fluorescence yield mode show that 2^nd^ and 5^th^ scales of onion have significantly higher calcium mass fractions than 8^th^ and 11^th^ scales (**Figures 2A, B**, **5A**). The numbering of the onion scales is such that 2^nd^ scale is the oldest and 11^th^ is the youngest of the peels examined in this study (Suslov et al., 2009; Kafle et al., 2014; Zhang et al., 2014). No significant difference is apparent in the calcium mass fraction between 8^th^ and 11^th^ scales. Both these younger scales are formed between 10 and 12 weeks of onion bulb development (Suslov et al., 2009), and they likely do not differ much from one another in structure and composition. Furthermore, a higher calcium mass fraction is found in bottom and middle regions in Arabidopsis hypocotyls than in the top region (**Figure 4C**; **Figures S3A, B**). For Arabidopsis hypocotyls, the top region is undergoing rapid cell expansion, such that the bottom region is more expanded tissue than the tissue at the top. This suggests that calcium composition in plant tissues might depend on the developmental stage of the tissue. Lower calcium concentration has been reported to be necessary for cell expansion (Bascom et al., 2018; Thor, 2019), such that the cells in inner/younger scales of onion and in the top region of the etiolated hypocotyls require lower calcium composition to allow for expansion and fast elongation (Gendreau et al., 1997; Suslov et al., 2009).

ICP-MS provides a quantitative measure of trace elements with high sensitivity, but it requires a much larger sample size than NEXAFS. In this study, 50 mg of dried onion peel was used for each ICP-MS measurement. In contrast, an onion peel of a few hundred microns weighing ~45 μg sufficed for a NEXAFS measurement, where the X-ray beam size for transmission NEXAFS was ~100 µm × 100 µm and for FY NEXAFS it was ~50 µm × 200 µm. Furthermore, X-ray spectroscopy to obtain elemental composition can be performed at high spatial resolution. Scanning Transmission X-ray Microscopy (STXM), which combines NEXAFS with imaging to provide chemically-sensitive images, has a spatial resolution that is approaching 10 nm, and has previously examined biological materials such as calcified coccolithophores (Vila-Comamala et al., 2009; Chao et al., 2012; Sun et al., 2014; Cosmidis et al., 2015). Here, NEXAFS data were collected from approximately the center of each region (top, middle, and bottom; each of length ~5 mm) of the hypocotyl and we could detect a significant change in relative calcium mass fraction between regions of Arabidopsis hypocotyls that are about 5 mm apart (**Figures 4B, C**).

Depending on the energies of incident X-rays, FY NEXAFS of plant samples will be limited in probe depth. The escape depth of photons for cell walls at the calcium K-edge (~4050 eV, tender regime) is ~7.3 to 48 μm and at the calcium L-edge (~350.0 eV, soft regime) is ~0.2 to 0.4 μm. Because the plant samples examined in this study are ~1 – 5 μm thick, FY NEXAFS performed at energies within the soft X-ray regime would not probe the entire thickness of the sample, while tender X-ray experiments would. Additionally, more artifacts are expected in correlating FY NEXAFS and ICP-MS results, because unlike in transmission NEXAFS, quantitative measure of the total absorption coefficient from FY spectra can be limited by self-absorption effects in thick and concentrated samples and the relative probability of an excited atom emitting electrons or photons (Hubbell et al., 1994; De Groot et al., 2010; Achkar et al., 2011). Nevertheless, the positive correlation between the edge jump in Ca K-edge FY NEXAFS and relative calcium mass fraction from ICP-MS (**Figure 5B**) suggests that FY NEXAFS is quantitative for studies of plant cell walls at the Ca K-edge.

Our NEXAFS results estimate 2.09 mg Ca/g of dry cell wall in 6-day-old etiolated Arabidopsis hypocotyl. Given that the uronic acid content is 800 nmol per mg of alcohol insoluble residue (which we assume is dry mass of hypocotyl cell wall) (Barnes et al., 2022), 86% of uronic acids are galacturonic acid (GalA) (Xiao et al., 2014), and 22.5% of GalA is methyl esterified (Barnes et al., 2022), our estimate of the calcium mass fraction corresponds to a molar ratio of calcium to GalA in the hypocotyls of approximately 1:10. For 5^th^ scale onion epidermal peels, the GalA content is 1.4 μmol/mg of dry onion (Wilson et al., 2021), 34% of GalA is methyl esterified (Wang et al., 2020), and the calcium mass fraction is 3 mg/g (**Figure 3B**), which leads to a 1:12 calcium to GalA ratio. The canonical egg-box model of calcium in the cell wall suggests a ratio of 1:4, indicating that the amount of calcium in the cell wall is smaller than the amount that can saturate all calcium-crosslinkable HG sites. Treating 5^th^ scale onion peels with 2 mM CaCl_2_ to saturate the cell wall leads to a calcium mass fraction of 9.9 mg/g and calcium to GalA ratio of 1:3.8, which is quantitative titration of the available calcium sites.

Although this work discusses the application of Ca NEXAFS to quantify calcium in dried onion epidermis and hypocotyls, the technique could be extended to live and complex tissues as well, such as roots, flowers, grains and leaves. NEXAFS has been used for characterization of complex biological tissues such as snake scales (Baio et al., 2015), frog tongue mucus (Fowler et al., 2018), and insect cuticle (Baio et al., 2019). STXM has also been used to study fully hydrated microbial biofilms (Lawrence et al., 2003). Moreover, the elemental Ca K-edge used in this study has been shown to be capable of characterizing calcium in solution (Zhang et al., 2015).

The X-ray beams used in this study were on the order of hundreds of microns in width, making measurements that are averaged over multiple cells. For measurements at the Ca L-edge, the technique remains limited by the sample thickness (<5 μm). This is less of a technical challenge at the Ca K-edge where the sample thickness can go up to 48 μm. Another challenge that can arise in applying this technique to different crop plants is finding the appropriate calibration sample for absolute quantification of calcium content because plant tissues vary widely in composition based on species, age, and environment.

In summary, we used NEXAFS in transmission mode at the calcium L-edge and NEXAFS in fluorescence yield mode at the calcium K-edge to quantify the calcium mass fraction in onion epidermal peels and etiolated hypocotyls of *Arabidopsis thaliana*. We normalize the transmission absorbance spectra at the calcium L-edge by the edge jump at the carbon K-edge to account for the sample thickness and obtain a quantitative measure of calcium composition. The calibration curve obtained from ICP-MS and NEXAFS measurements of onion epidermal peels was used to estimate the calcium mass fraction in hypocotyls of *Arabidopsis thaliana*, which have similar cell wall compositions as onion samples. Thus, NEXAFS provides an approach to measure calcium levels in plant samples, which is critical for plant physiological processes and for technological development in agriculture, food, and forest industries.

## Data availability statement

The original contributions presented in the study are included in the article/**Supplementary Material**. Further inquiries can be directed to the corresponding authors.

## Author contributions

SR, EWG and EDG designed the project. WB and OS grew Arabidopsis hypocotyls. SR, WB, OS, and JD prepared samples for experiments. SR, OS, JD, CW, GF, and MZ designed and carried out X-ray experiments. WB and CA designed and carried out immunolabeling experiments. OS designed and carried out ICP-MS experiments. All authors contributed to analyses of data and writing and editing of the text. All authors contributed to the article and approved the submitted version.
