# Supplementary material for: Measuring calcium content in plants using NEXAFS spectroscopy
Source: Front Plant Sci. 2023 Aug 16;14:1212126. doi: 10.3389/fpls.2023.1212126 (PMC10468975; doi:10.3389/fpls.2023.1212126)
Supplement: Supplementary file 1 [file DataSheet_1.pdf]

**Supplementary Information for**

# Measuring calcium content in plants using NEXAFS spectroscopy

*Sintu Rongpipi,[1] William J. Barnes,[2] Oskar Semianowski,[2] Joshua T. Del Mundo,[1] Cheng Wang,[3] Guillaume Freychet,[4] Mikhail Zhernenkov,[4] Charles T. Anderson,[2] Esther W. Gomez,[1,5*] and Enrique D. Gomez[1,6*]*

[1]Department of Chemical Engineering, The Pennsylvania State University, University Park, PA 16802, United States

[2]Department of Biology, The Pennsylvania State University, University Park, PA 16802, United States

[3]Advanced Light Source, Lawrence Berkeley National Laboratory, 1 Cyclotron Road, Berkeley, CA 94720, United States

[4]NSLS-II, Brookhaven National Laboratory, Upton, New York 11973, United States

[5]Department of Biomedical Engineering, The Pennsylvania State University, University Park, PA 16802, United States

[6]Department of Materials Science and Engineering and Materials Research Institute, The Pennsylvania State University, University Park, PA 16802, United States

*edg12@psu.edu, ewg10@psu.edu

**Supplementary Section 1:** Calculation of the thickness of primary cell wall samples from NEXAFS

The thickness of a cell wall sample can be estimated from transmission NEXAFS using the absorbances at off-edge energies. The thickness of a measured sample is related to the absorbance (Abs) as described in equation 2 in the Results section:

$$Abs = \mu \rho t$$

where $\mu$ is the mass absorption coefficient, $\rho$ is the density, and $t$ is the thickness of the sample. Thus, the thickness can be estimated as:

$$t = \frac{Abs}{\mu \rho}$$

Assuming that the $\mu$ and $\rho$ of primary cell walls remain invariant in the different cell wall samples, we normalize the background corrected calcium L-edge absorbance by absorbance at off-edge energies to obviate the effects of sample thickness on NEXAFS data.

**Supplemental Section 2:** We systematically varied the background subtraction for the Ca L-edge NEXAFS data to achieve the expected linear trend for the pre-edge (~340-345 eV) and post-edge (~355-360 eV). Thus, we examined 7 different baseline fit options as follows:

1. Linear baseline fit pre-edge 340 – 342 eV
2. Linear baseline fit pre-edge 335 – 345 eV
3. Linear baseline fit pre-edge 335 – 340 eV
4. Linear baseline fit pre-edge 340 – 345 eV
5. Linear baseline fit post-edge 355 – 360 eV
6. Linear baseline fit post-edge 355 – 365 eV
7. Cubic baseline fit pre-edge 335 – 345 eV

From Figures S1a-d, we see that subtracting linear baselines from pre-edge regions 335-345 eV and 335-340 eV lead to a high post edge background that becomes more evident in younger onion scales ($8^{th}$ and $11^{th}$) where calcium content is lower. Using a background subtraction from a linear fit to the 340-345 eV region leads to negative post-edge spectra at some energies, which is unphysical. Similarly, subtracting a background from post-edge regions (355 to 360 eV or 355 to 365 eV) or using a cubic baseline fit to subtract the background (fit to pre-edge at 335-345 eV) also leads to negative values for the spectra. Thus, from the various options for background subtraction explored for this work, fitting a linear function to the 340-342 eV pre-edge region leads to reasonable spectra with minimal contributions from background artefacts.

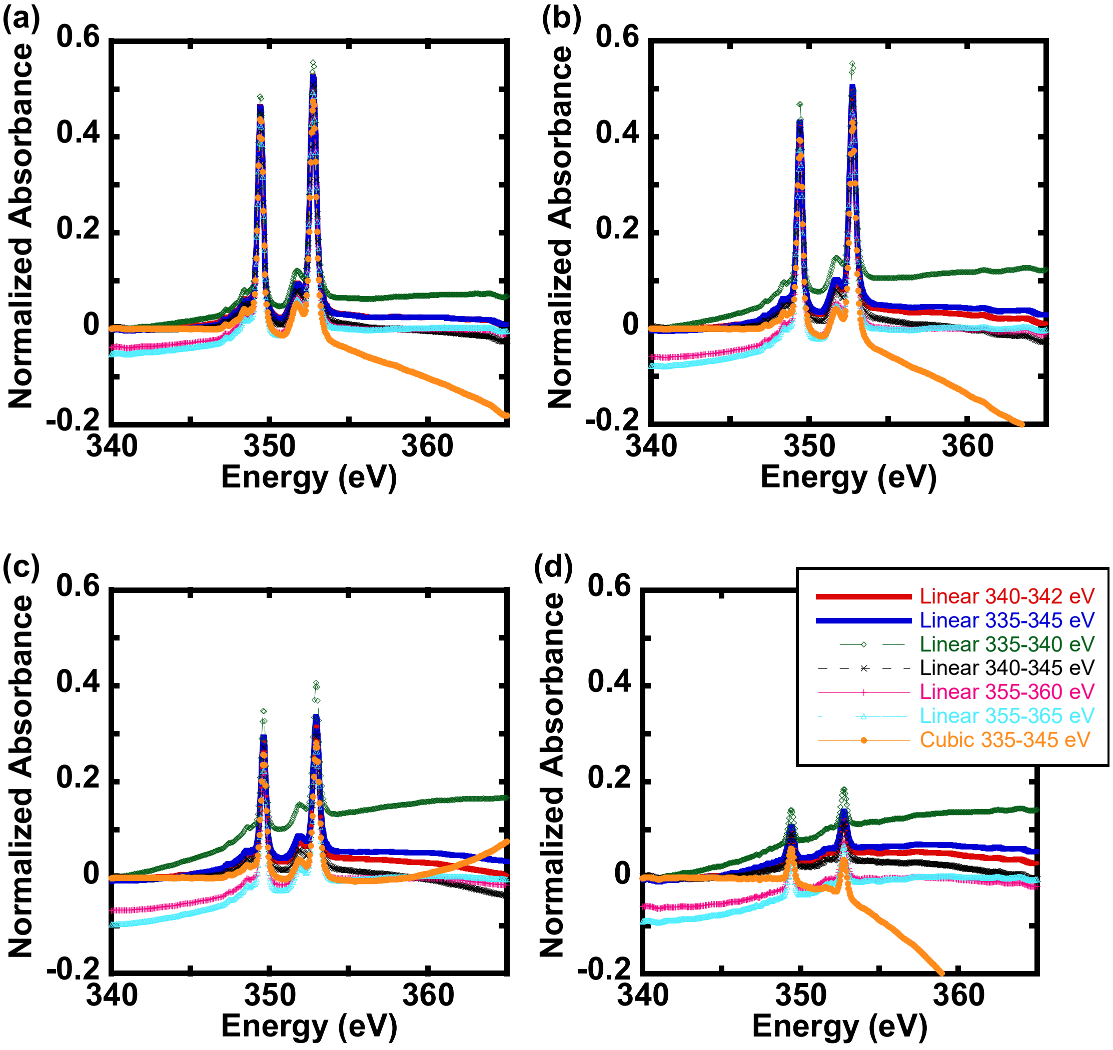


**Supplemental Figure 1:** Absorbance at the calcium $L_{3,2}$ edge of unextracted (a) 2nd, (b) 5th, (c) 8th, and (d) 11th onion epidermal cell walls. Spectra are subtracted by a background obtained from linear baseline fit pre-edge 340 – 342 eV (red), linear baseline fit pre-edge 335 – 345 eV (dark blue), linear baseline fit pre-edge 335 – 340 eV (green), linear baseline fit pre-edge 340 – 345 eV (black), linear baseline fit post-edge 355 – 360 eV (pink), linear baseline fit post-edge 355 – 365 eV (light blue), and cubic baseline fit pre-edge 335 – 345 eV (orange).

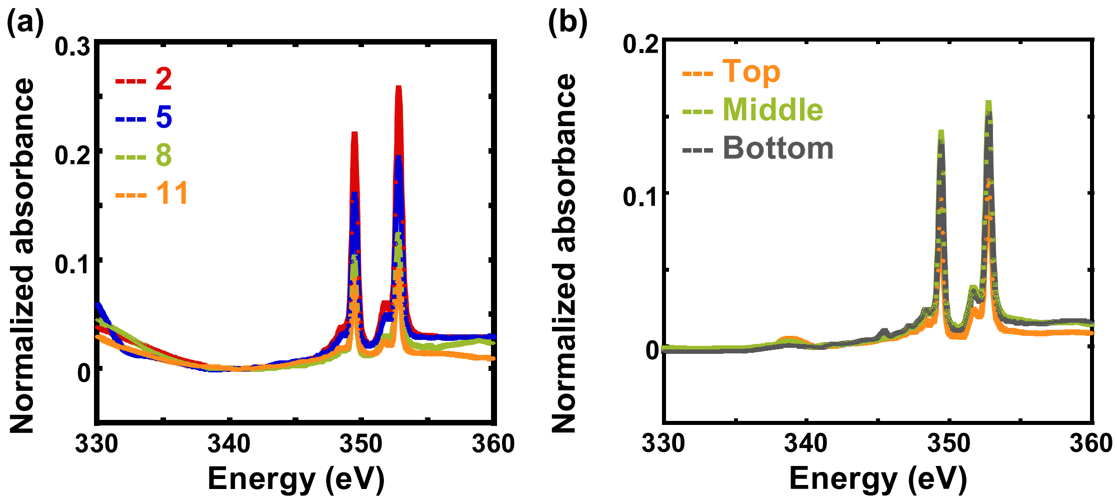


**Supplementary Figure 2:** (a) Absorbance at the calcium $L_{3,2}$ edge of unextracted $2^{nd}$, $5^{th}$, $8^{th}$, and $11^{th}$ onion epidermal cell walls. (b) Absorbance at the calcium $L_{3,2}$ edge (330.0 eV to 360.0 eV) of the top, middle, and bottom regions of 6-day-old hypocotyls of *Arabidopsis thaliana.* All spectra are corrected by a linear background obtained from the pre-edge (340.0 to 342.0 eV) as described in the Methods and normalized by the carbon edge step jump ($\text{Absorbance}_{325.0\ \text{eV}} - \text{Absorbance}_{270.0\ \text{eV}}$).

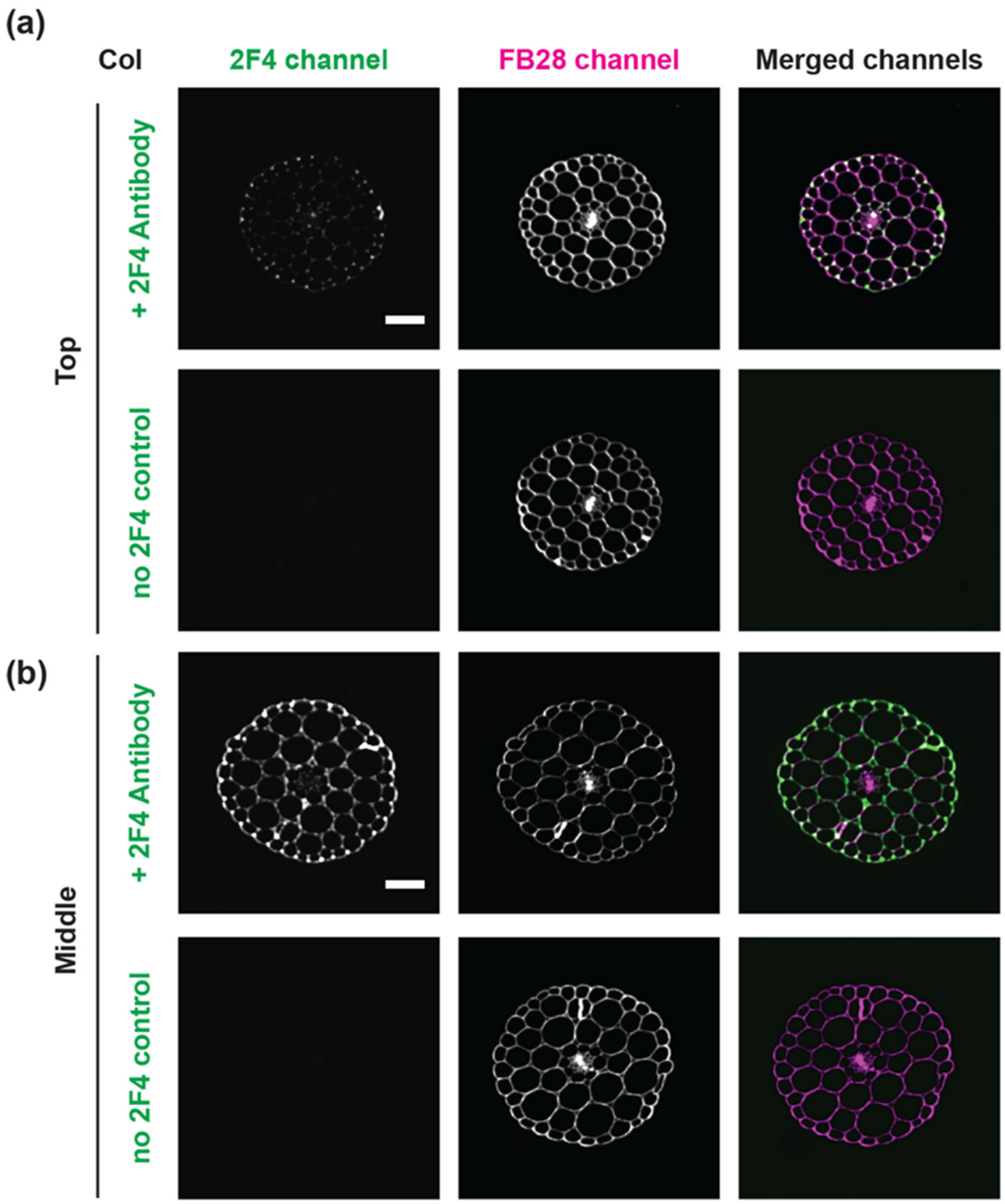


**Supplementary Figure 3:** 2F4 immunolabeling of $Ca^{2+}$-crosslinked homogalacturonan (HG) is more apparent in the middle region of etiolated 6-day-old Col-0 seedling hypocotyls compared to the top region. (a) Maximum projections of 2F4 (recognizes $Ca^{2+}$-crosslinked HG) and FB28 (a dye that binds to glucans) co-labeling in cross-sections derived from the top of hypocotyls of 6-day-old etiolated Col-0 seedlings (top row). A no primary antibody (2F4) negative control is shown in the bottom row. (b) Maximum projections of 2F4 and FB28 co-labeling in cross-sections derived from the middle of hypocotyls of 6-day-old etiolated Col-0 seedlings (top row). A no primary antibody (2F4) negative control is shown in the bottom row. (a,b) All 2F4 signals were contrast enhanced to the same Lookup Table (LUT) for accurate comparison. FB28 signals were separately enhanced for each merged image to depict the cell wall. Scale bars: 100 µm.

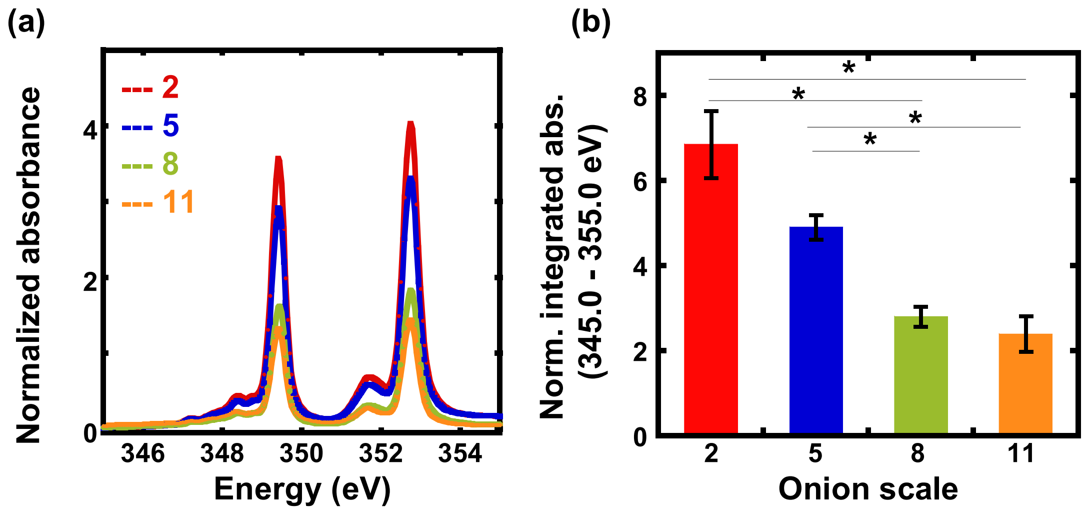


**Supplementary Figure 4:** (a) Absorbance near the calcium $L_{3,2}$ edge of unextracted 2$^{nd}$, 5$^{th}$, 8$^{th}$, and 11$^{th}$ onion epidermal cell walls. Spectra are corrected by a linear background obtained from the pre-edge (340.0 to 342.0 eV) as described in the Methods and then normalized by the average absorbance for off-edge energies from 325.0 eV to 330.0 eV. (b) Background corrected absorbance normalized by off-edge absorbances (for energies 325.0 eV to 330.0 eV), integrated between 345.0 eV to 355.0 eV for unextracted onion epidermal cell walls. Error bars represent standard error of the mean and asterisks indicate significant differences (n=3, $^*p < 0.01$).

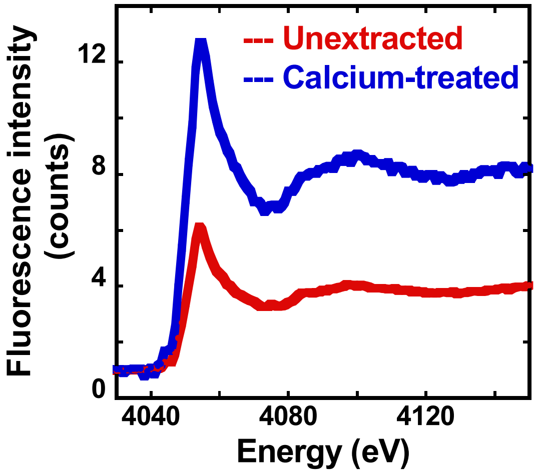


**Supplementary Figure 5:** Fluorescence NEXAFS spectra (acquired in a reflection geometry) for unextracted and calcium-treated onion 5$^{th}$ scale at the calcium K-edge. Spectra are normalized by the average of the Fluorescence Yield intensities at off-edge energies (4030 eV – 4040 eV).

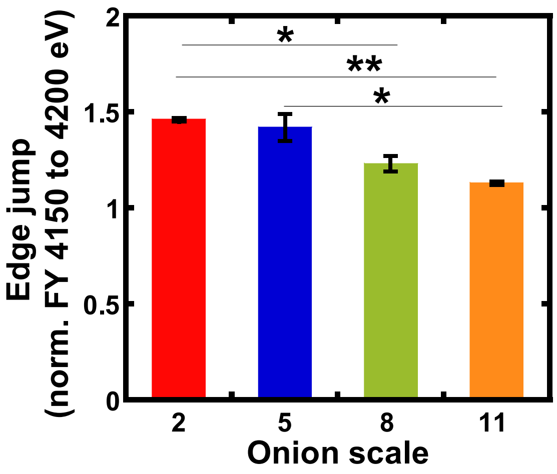


**Supplementary Figure 6:** Edge jump (average of intensities for post-edge energies 4150 eV to 4200 eV from FY spectra normalized by average of off-edge intensities 4030 eV to 4040 eV) of unextracted onion scales. Error bars represent standard error of the mean and asterisks indicate significant differences (n=3, *p < 0.05 **p<0.01).
